# First-Year Outcomes of Cataract Surgery Combined with Intravitreal Ranibizumab Injection in Wet Age-Related Macular Degeneration

**DOI:** 10.4274/tjo.galenos.2018.76429

**Published:** 2019-02-28

**Authors:** Sabahattin Sül, Aylin Karalezli, Müjdat Karabulut

**Affiliations:** 1 Muğla Sıtkı Koçman University Faculty of Medicine, Department of Ophthalmology, Muğla, Turkey

**Keywords:** Neovascular age-related macular degeneration, ranibizumab, cataract surgery

## Abstract

**Objectives:**

To compare the first-year results of patients with active neovascular age-related macular degeneration (nAMD) under intravitreal ranibizumab (IVR) treatment who did and did not undergo cataract surgery.

**Materials and Methods:**

The records of 72 patients with active nAMD were reviewed retrospectively. Group 1 consisted of 23 patients who underwent uncomplicated cataract surgery and continued with IVR treatment and group 2 consisted of 49 patients without cataract who received only IVR treatment. The groups were compared according to pretreatment and first year best spectacle-corrected visual acuity (BCVA), central foveal thickness (CFT), number of injections, and nAMD activity (presence of subretinal or intraretinal fluid). Logarithm of minimum angle of resolution (LogMAR) was used for the determination of visual acuity. Activity findings were evaluated with optical coherence tomography.

**Results:**

Pretreatment BCVA was 0.94±0.21 in group 1 and 0.77±0.36 in group 2 (p=0.041). At the end of the first year, BCVA was 0.48±0.35 in group 1 and 0.49±0.33 in group 2 (p=0.902). BCVA change was 0.46±0.29 in group 1 and 0.28±0.31 in group 2 (p=0.026). Pretreatment CFT was 305±146 μm in group 1 and 340±120 μm in group 2 (p=0.292). At the end of the first year, CFT was 246±110 μm and 245±82 μm in group 2 (p=0.977). CFT change was 59±45 μm in group 1 and 92±97 μm in group 2 (p=0.135). Mean number of injections over 1 year was 6.2±1.9 in group 1 and 5.7±1.8 in group 2 (p=0.271). At the end of the first year, subretinal fluid was observed in 3 patients in group 1 (13%) and 5 patients in group 2 (10.2%) (p=0.721) and intraretinal fluid was present in 3 patients in group 1 (13%) and 4 patients in group 2 (8.2%) (p=0.515).

**Conclusion:**

Cataract surgery combined with IVR treatment yielded significant visual gain in patients with active nAMD. Anatomic results suggest that cataract surgery does not worsen nAMD.

## Introduction

Age-related macular degeneration (AMD) and cataract are the main causes of vision loss in the elderly population.^1,2^ With longer average life expectancy in developed countries, the incidence rates of AMD and cataract are increasing. Today, treatment of neovascular AMD with anti-vascular endothelial growth factors (anti-VEGFs) stabilizes or increases vision in most patients.^3,4^ The timing of cataract surgery is important in AMD patients; there is still debate regarding the effect of cataract surgery on AMD progression. Although some authors have reported that cataract surgery encourages AMD progression, others studies showed that cataract surgery had no effect on the progression of AMD.^5,6,7,8,9^

The aim of this study was to evaluate and compare 1-year visual and anatomic outcomes in patients with active neovascular AMD treated with intravitreal anti-VEGF therapy who did and did not undergo cataract surgery.

## Materials and Methods

We retrospectively evaluated the records of patients with active neovascular AMD with or without cataract who received intravitreal ranibizumab (IVR) therapy (0.5 mg/0.05 mL) with or without uneventful phacoemulsification surgery in 2016-2017. Ethical approval for the study was obtained from the Ethics Committee of Muğla Sıtkı Koçman University Medical Faculty and the study was conducted in accordance with the Declaration of Helsinki. Consent forms were obtained from all patients before each intraocular injection and cataract surgery.

The patients were divided into two groups, those with nAMD + cataract who underwent both cataract surgery and IVR therapy (group 1) and phakic patients with nAMD but no cataract who underwent IVR therapy only (group 2). Age, sex, best corrected visual acuity (BCVA), complete ophthalmologic examination records, fundus fluorescein angiography (FA), and optical coherence tomography (OCT) measurements were recorded before treatment in both groups. All patients received 3 monthly injections of 0.5 mg IVR initially, after which IVR injections were repeated as needed. Repeat injections were given to patients with intraretinal and/or subretinal fluid in OCT. Patients who had no signs of disease activity and had not received IVR treatment for at least 3 months but showed activation at the last visit were included in the study. IVR injection and cataract surgery were combined in patients in group 1. Patients with visual loss associated with other retinal (retinal vascular pathologies) or corneal pathologies and patients with choroidal neovascularization not related to AMD were excluded from the study. All patients were followed monthly for 1 year. Visual and anatomic response at 1 year and total injection numbers were evaluated and compared between the groups. Pre- and post-treatment visual acuities were measured using Snellen chart and converted to LogMAR for statistical analysis. Central foveal thickness (CFT), presence of subretinal or intraretinal fluid (increase, decrease, or no change), and subfoveal choroidal thickness were used to evaluate and compare anatomic response.

### Statistical Analysis

SPSS (version 22.0) statistical program was used to evaluate the study data. T-test and chi-square test were used in comparisons of continuous and categorical variables between the groups, respectively. P<0.05 was considered statistically significant.

## Results

Seventy-two eyes of 72 patients were included in the study. Group 1 included 23 eyes and group 2 included 49 eyes. Mean age was 72.1±5.1 (64-85) years in group 1 and 74.3±6 (62-87) years in group 2 (p=0.151). Male:female ratios were 14:9 (60.9%/39.1%) in group 1 and 37:12 (75.5%/24.5%) in group 2 (p=0.203). The change from pre-treatment to 1-year BCVA was significantly greater in group 1 than in group 2 (Table 1). There was no significant difference between the groups in 1-year BCVA (Table 1). At 1 year, visual acuity was unchanged in 3 patients (13%) and decreased in 1 patient (4.3%) in group 1. In group 2, visual acuity was unchanged in 10 patients (20.4%) and decreased in 4 patients (8.2%). Change in CFT was 59±45 µm in group 1 and 92±97 µm in group 2 (p=0.135). Pre-treatment and 1-year CFT values were similar (Table 1). Mean number of injections was 6.2±1.9 in group 1 and 5.7±1.8 in group 2 (p=0.271) (Table 1). The prevalence of subretinal exudation was similar in groups 1 and 2 before treatment (Table 2). At 1 year, subretinal exudation was detected in 3 patients (13%) in group 1 and 5 patients (10.2%) in group 2 (p=0.721) and intraretinal cyst was observed in 3 patients (13%) in group 1 and 4 patients (8.2%) in group 2 (p=0.515).

## Discussion

Cataract and AMD are common in the elderly population and their coexistence can compound visual impairment in this age group. There is no consensus regarding what treatment will provide maximum visual acuity in such cases. It is clear that the visual gains achieved with ongoing AMD treatment will be obscured by the presence of cataract. However, there is still debate concerning the effect of cataract surgery timing on the progression of AMD. In earlier studies on this subject it was reported that progression to advanced AMD was more rapid after cataract surgery. In the Beaver Dam Eye study, patients with early dry AMD showed a higher rate of advanced AMD (geographic atrophy or exudative form) after cataract surgery.^10^ Cugati et al.^11^ reported that patients who underwent cataract surgery had a 3.4-fold higher risk of developing choroidal neovascularization. The Rotterdam study also suggested that geographic atrophy was more common after cataract surgery.^2^ In a study of patients with bilateral symmetric early AMD, Pollack et al.^5^ found that eyes subjected to extracapsular cataract extraction and intraocular lens implantation showed higher incidence of wet AMD compared to unoperated eyes (19.1% in operated eyes vs. 4.3% in unoperated eyes). The Blue Mountains Eye, Beaver Dam Eye, and other population-based studies conducted before the anti-VEGF era included patients with dry AMD. The conclusion reached in these earlier studies that cataract surgery has a negative effect on AMD progression may be related to several factors. The first is that these studies compared patients with dry AMD with healthy elderly individuals. Considering the natural course of the disease, higher rates of nAMD development can be expected in some patients compared to healthy individuals. Wang et al.^12^ conducted a clinical study comparing fellow eyes of AMD patients and found that cataract surgery had no effect on progression, while the presence of early AMD was the most important risk factor for the development of advanced AMD. Other factors may include inadequate AMD diagnosis or staging due to lens opacity and the use of extracapsular cataract extraction instead of modern phacoemulsification surgery (due to the more sudden change in intraocular pressure and probably more severe postoperative inflammation compared to phacoemulsification). More recent studies have not detected any association between cataract surgery and AMD progression. The Age-Related Eye Disease Study 2 study demonstrated significantly increased visual acuity after surgery.^13^ In a randomized controlled trial in which FA was performed before and 6 months after surgery, nAMD was observed in only 1 of 27 patients who underwent surgery and none of the 29 patients who did not have surgery.^14^ There was also a significant increase in visual acuity in the operated group. Akıncı and Yalnız^15^ showed that cataract surgery had no effect on AMD progression in their 15 patients.

Before the anti-VEGF era, cataract surgery was not recommended in patients with neovascular AMD. It was believed that the inflammation, blood-retinal barrier disruption, and intraocular pressure changes resulting from surgery would lead to decompensation in the fragile choroidal vessels, thus causing further leakage and hemorrhage. However, since the introduction of anti-VEGF therapy, the effectiveness of anti-VEGFs against choroidal neovascularization and the better control of intraocular pressure provided by phacoemulsification have allowed cataract surgery to be performed more safely. In studies reported in the literature, cataract surgery is performed in neovascular AMD patients receiving anti-VEGF therapy. In a study by Furino et al.^,17^ 20 patients with active neovascular AMD and cataract underwent phacoemulsification with intravitreal bevacizumab administered at the end of surgery. The authors reported a significant increase in visual acuity and a significant decrease in central retinal thickness after 1 month. Tabandeh et al.^18^ reported that cataract surgery improved visual acuity in patients receiving anti-VEGF therapy with no increase in reactivation during follow-up.In their retrospective controlled study, Saraf et al.^19^ also determined that cataract surgery improved visual acuity and was not associated with worsening AMD. In addition, they reported greater retinal thickness in the operated eyes and stated that surgery in these eyes could cause a predisposition for cystoid macular edema. In our study, similar visual acuity was achieved in both groups at 1 year, with comparable numbers of patients with increased BCVA (82.7% in group 1 and 71.6% in group 2). Visual acuity was lower initially in the first group due to cataract; therefore, they achieved significantly greater visual gains compared to group 2 as a result of cataract surgery. At 1 year, the patients who underwent cataract surgery had received 0.5 more injections on average compared to patients without cataract, but the difference in injection numbers was not statistically significant. This suggests that any possible reactivation of neovascular vessels due to cataract surgery can be controlled with ranibizumab. When anatomic responses were evaluated, the rate of subretinal exudation was similar between the two groups. However, the patients who underwent cataract surgery showed higher rates of intraretinal cyst and smaller decrease in retinal thickness, although the differences were not statistically significant. Based on the aforementioned studies and our study, anti-VEGF therapy after cataract surgery halts and reverses active exudation and increases visual acuity in patients with neovascular AMD. 

Cataract surgery was reported to cause an increase in choroidal thickness.^20^ The drop in intraocular pressure and rise in ocular perfusion pressure accompanied by increased choroidal thickness may be associated with increased inflammation due to elevated prostaglandin and cytokines, which are thought to cause cystoid macular edema in the retina.^19,20,21,22^ However, in our study, there was no significant difference in choroidal thickness between the patients who did and did not have cataract surgery, and choroidal thickness decreased in both groups due to anti-VEGF use.

## Conclusion

In conclusion, cataract surgery provides significant visual improvement in patients with active AMD. No unfavorable impact on AMD progression was observed in eyes that underwent cataract surgery while receiving anti-VEGF therapy. Combined cataract surgery and anti-VEGF therapy can be considered an effective and reliable treatment modality in patients with active neovascular AMD.

## Figures and Tables

**Table 1:**
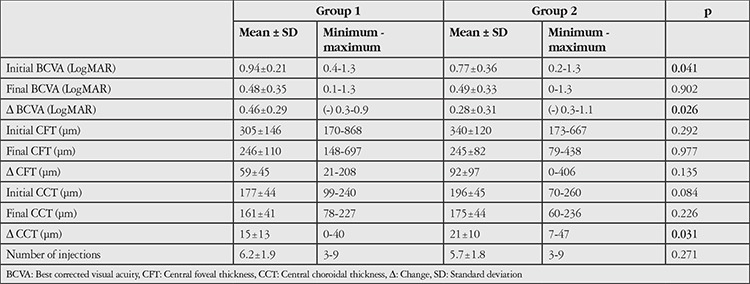
Visual and anatomic characteristics and comparison of injection numbers in age-related macular degeneration patients with cataract (group 1) and without cataract (group 2) before and after treatment

**Table 2:**
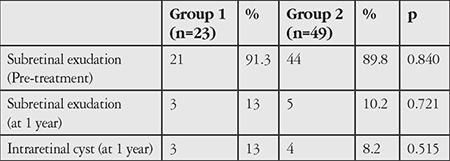
Comparison of rates of subretinal exudation and intraretinal cyst before and after treatment in age-related macular degeneration patients who had cataract surgery (group 1) and those without cataract (group 2)
